# A Case of Pulmonary Hamartoma Showing Rapid Growth

**DOI:** 10.1155/2013/231652

**Published:** 2013-09-19

**Authors:** Masamichi Itoga, Yoshiki Kobayashi, Masahide Takeda, Yuki Moritoki, Mami Tamaki, Kenji Nakazawa, Toru Sasaki, Hayato Konno, Ikuo Matsuzaki, Shigeharu Ueki

**Affiliations:** ^1^Department of Infection, Allergy, Clinical Immunology and Laboratory Medicine, Akita University Graduate School of Medicine, 1-1-1 Hondo, Akita 010-8543, Japan; ^2^Department of Otolaryngology, Kansai Medical University, 2-5-1 Shin-machi, Hirakata City, Osaka 573-1010, Japan; ^3^Japan Labour, Health and Welfare Organization, Akita Rosai Hospital, 30 Karuizawa Shimotai, Odate City, Akita 018-5604, Japan; ^4^Noshiro Yamamoto Medical Association Hospital, 105-11 Hiyama Shin-tazawa, Noshiro City, Akita 016-0151, Japan

## Abstract

A 65-year-old man was admitted for detailed examination of a growing nodular shadow in the left lung. The nodular shadow was initially detected in a routine chest X-ray check-up in March 2012 that warranted regular chest X-ray follow-up. The nodular shadow increased in size from 12 × 15 mm to 15 × 20 mm within five months. The calculated tumor doubling time (TDT) in our case was approximately 132.2 days. A malignant tumor was strongly suspected based on the rapid growth, and tumorectomy was thus performed. Cartilaginous tissue accounted for most of the pathological specimen, but a small amount of an epithelial component was observed histologically, and we diagnosed a hamartoma. Hamartoma generally shows slow annual growth, but it is important to recognize that rapid enlargement occurs in some cases.

## 1. Introduction

Hamartomas, introduced by Albrecht in 1904 [1], are tumor-like malformations due to abnormal mixing or abnormal development of normal tissue components of the organ in which they occur. This abnormality is thought to be the result of variations in the quality, arrangement, or degree of differentiation of the tissues [2]. Hamartoma is a frequent disease, accounting for 77% of all benign lung tumors. The majority of pulmonary hamartomas are asymptomatic and show slow annual growth [3–5]. However, it is also important to recognize that some hamartomas might increase rapidly in size [6] and show malignant alteration [7, 8]. Here, we report a case with a hamartoma that was difficult to differentiate from lung cancer due to its rather rapid enlargement.

## 2. Case Report

**Figure 1:**
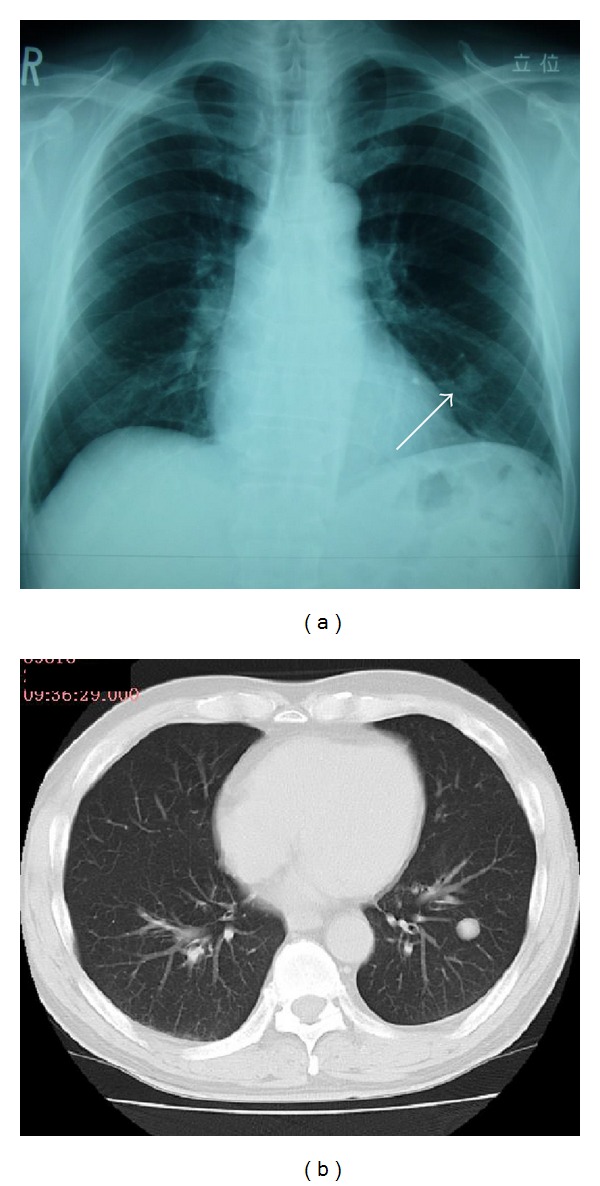
Chest X-ray and CT findings on admission. (a) Simple chest X-ray indicated a smooth, well-circumscribed nodule in the left lower lung field (white arrow). (b) Simple chest computed tomography revealed a nodule measuring 17 mm in diameter and having a well-defined border in S9 of the left lung.

A 65-year-old man was informed of a nodular shadow of the left lung when he received a routine chest X-ray check-up in March 2012. After 5-month follow-up by chest X-ray, he was admitted for detailed examination of the growing nodular shadow in the left lung, which had increased in size from 12 × 15 mm to 15 × 20 mm (Figure 1(a)).

On admission, he was asymptomatic. His family history was unremarkable. He had smoked 20 cigarettes per day for 50 years. Physical examination, laboratory tests, and tumor marker measurements revealed no abnormalities. In addition, serum *Cryptococcus *antigen was negative. Plain computed tomography (CT) of the chest revealed a nodule measuring about 20 mm in diameter with a well-defined border and internal heterogeneity in the S9 area of the left lung (Figure 1(b)). On fluorodeoxyglucose (FDG) positron emission tomography (PET), no abnormal FDG accumulation was detected in the left lower lobe nodule, raising the possibility of a well-differentiated tumor or a small cell carcinoma. Sputum cytology did not detect any atypical cells. Examination results for septum bacteria and acid-fast bacilli were negative.

**Figure 2:**
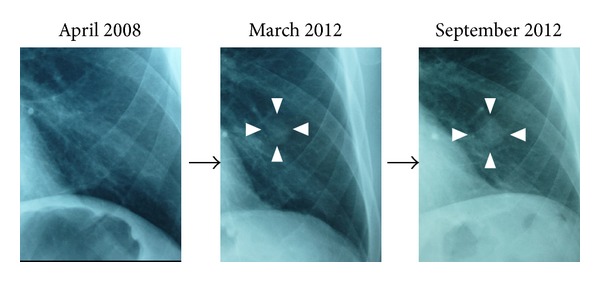
Change in a diachronic nodule shadow. A growing nodular shadow in the left lower lung field is indicated in the white arrow heads. The nodule had increased in size from 12 × 15 mm (March 2012) to 15 × 20 mm (September 2012), whereas no nodular shadows are detectable in April 2008.

Bronchoscopic examination was performed on admission. No abnormalities were recognized in the observed area. The results of bronchial brushing and bronchial lavage cytology were class II and negative for malignancy. Although bronchoscopic examination failed to yield a definitive diagnosis, a malignant tumor was strongly suspected based on the rapid growth (Figure 2). CT-guided percutaneous fine-needle aspiration biopsy was not considered to avoid the risk of iatrogenic pneumothorax as he had a 50-pack-year smoking history with scattered low attenuation areas in the lung fields. To make an exact diagnosis, left lung segmental resection was performed via thoracoscopy.

**Figure 3:**
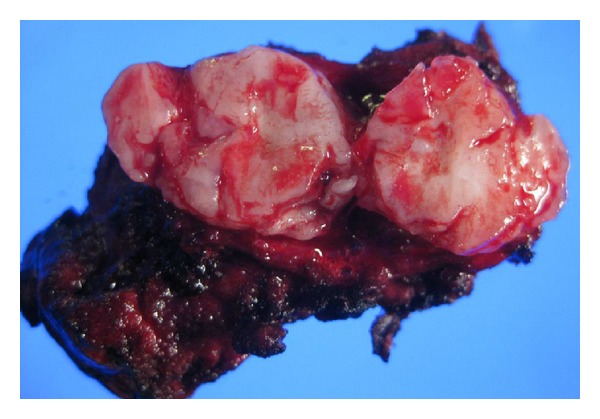
Photograph illustrating the cut surface of the resected pulmonary hamartoma. The tumor was about 20 mm in the greatest dimension and was well circumscribed.

**Figure 4:**
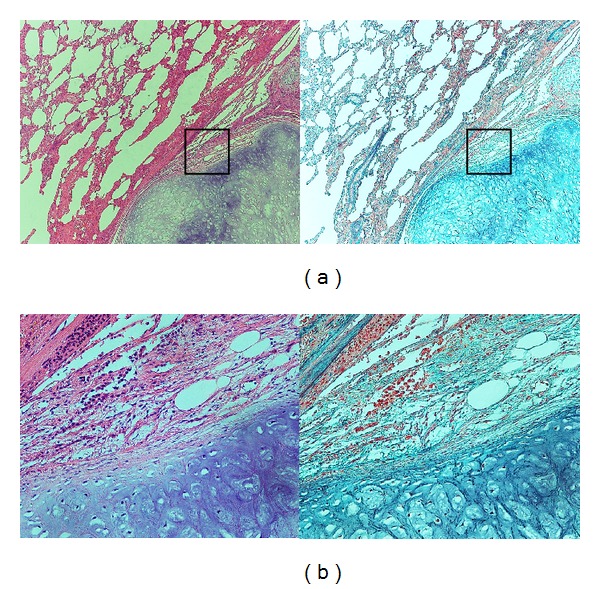
(a) Histology of the pulmonary hamartoma. The section showing the typical appearance of the pulmonary hamartoma. A lobular mass of mature hyaline cartilage is intimately admixed with fibromyxoid stroma. (Left: Hematoxylin-Eosin stain; ×40. Right: Elastica-Masson stain; ×40). (b) Higher magnification of black box in (a) (Left: Hematoxylin-Eosin stain; ×200. Right: Elastica-Masson stain; ×200).

Gross examination of the specimen disclosed a lobular, well-circumscribed, and yellowish-white mass about 20 mm in diameter (Figure 3). Histologically, a lobular mass of mature hyaline cartilage was intimately admixed with fibromyxoid stroma (Figure 4). Therefore, malignancy was ruled out, and we diagnosed a hamartoma. Six months after surgical resection, the patient remains well with no recurrence.

## 3. Discussion

Hamartoma is a clinically frequent benign lung tumor, accounting for 77% of all benign lung tumors [3]. As for the radiographic characteristics, they are typically smooth, well-circumscribed nodules, round or oval, with heterogeneous internal structures. Popcorn-like calcification may also be observed. The frequency of calcification was reported to be approximately 30% [9]. FDG-PET is useful for the differentiation of pulmonary mass lesions. In our case, because there was no abnormal accumulation of FDG, we were able to judge the lesion to very likely be a benign tumor. However, some reports on hamartomas describe difficulty with differentiation from malignant tumors when slight abnormal accumulation of FDG is recognized [10].

Hamartoma is considered to be a benign tumor with a good prognosis and often followed up by observation without surgery due to slow annual growth. The mean transverse diameter of hamartomas at discovery was reported to be 21.7 ± 16.2 mm, and the average increase in transverse diameter was 3.2 ± 2.6 mm per year [4]. By converting the rate of increase to tumor doubling time (TDT) as an objective index, the average TDT of hamartomas was calculated to be 581.2 days [11]. In our case, the TDT was 132.2 days, much shorter than that in previous reports. Hasegawa et al. reported the TDT of pulmonary tumors to be 97 ± 46 days for small cell carcinomas, 129 ± 97 days for squamous cell carcinomas, and 533 ± 381 days for adenocarcinomas [12]. In addition, Usuda et al. reported the TDT of pulmonary tumors to be 79.4 ± 51.9 days for large cell carcinomas, 80.8 ± 49.7 days for small cell carcinomas, 104.7 ± 105.6 days for squamous cell carcinomas, and 223.1 ± 209.4 days for adenocarcinomas [13]. Our case can be regarded as relatively rare with a TDT equivalent to that of squamous cell carcinomas in earlier reports, which made it difficult to differentiate from lung cancer.

As with our case, some cases showing rapid growth have been reported [6]. Furthermore, some reports presented cases of hamartoma with malignant alteration [7, 8]. Okabayashi et al. presented a case of a giant pulmonary hamartoma with a high production of carbohydrate antigen (CA) 19-9 [14]. A molecular biological search approach revealed a relationship between hamartoma and abnormalities of 12q14 and 6q21, domains encoding the high mobility group A (HMGA) genes, which are important for controlling chromosome structures [15]. Taken together with the fact that the incidence rate of lung cancer in pulmonary hamartoma is reported to be 6.6 times higher than in healthy subjects [16], pulmonary segmental resection is recommended for hamartomas with a neoplastic nature rather than enucleation [17]. Therefore, pulmonary segmental resection was performed as diagnostic therapy in our case.

## 4. Conclusion

We have presented a case with a pulmonary hamartoma requiring differentiation from lung cancer due to a relatively rapid increase in size. It is of note that hamartomas generally show relatively slow annual growth, but rapid enlargement occurs in some cases.
